# Layered and integrated medical countermeasures against *Burkholderia pseudomallei* infections in C57BL/6 mice

**DOI:** 10.3389/fmicb.2022.965572

**Published:** 2022-08-17

**Authors:** Christopher P. Klimko, Jennifer L. Shoe, Nathaniel O. Rill, Melissa Hunter, Jennifer L. Dankmeyer, Yuli Talyansky, Lindsey K. Schmidt, Caitlyn E. Orne, David P. Fetterer, Sergei S. Biryukov, Mary N. Burtnick, Paul J. Brett, David DeShazer, Christopher K. Cote

**Affiliations:** ^1^Bacteriology Division, United States Army Medical Research Institute of Infectious Diseases, Frederick, MD, United States; ^2^Department of Microbiology and Immunology, University of Nevada, Reno School of Medicine, Reno, NV, United States; ^3^Biostatistics Division, United States Army Medical Research Institute of Infectious Diseases, Frederick, MD, United States; ^4^Department of Microbiology and Immunology, Faculty of Tropical Medicine, Mahidol University, Bangkok, Thailand

**Keywords:** *Burkholderia pseudomallei*, melioidosis, vaccine, antibiotics, mice, aerosols

## Abstract

*Burkholderia pseudomallei*, the gram-negative bacterium that causes melioidosis, is notoriously difficult to treat with antibiotics. A significant effort has focused on identifying protective vaccine strategies to prevent melioidosis. However, when used as individual medical countermeasures both antibiotic treatments (therapeutics or post-exposure prophylaxes) and experimental vaccine strategies remain partially protective. Here we demonstrate that when used in combination, current vaccine strategies (recombinant protein subunits AhpC and/or Hcp1 plus capsular polysaccharide conjugated to CRM197 or the live attenuated vaccine strain *B. pseudomallei* 668 Δ*ilvI*) and co-trimoxazole regimens can result in near uniform protection in a mouse model of melioidosis due to apparent synergy associated with distinct medical countermeasures. Our results demonstrated significant improvement when examining several suboptimal antibiotic regimens (e.g., 7-day antibiotic course started early after infection or 21-day antibiotic course with delayed initiation). Importantly, this combinatorial strategy worked similarly when either protein subunit or live attenuated vaccines were evaluated. Layered and integrated medical countermeasures will provide novel treatment options for melioidosis as well as diseases caused by other pathogens that are refractory to individual strategies, particularly in the case of engineered, emerging, or re-emerging bacterial biothreat agents.

## Introduction

*Burkholderia pseudomallei* is a gram-negative bacterium that causes the disease melioidosis (Galyov et al., 2010; Brett et al., 2018). *B. pseudomallei* has been identified in Southeast Asia, northern Australia, and in many other tropical areas around the world (Dance, 2000; Aardema et al., 2005; Inglis et al., 2006; Lo et al., 2009; Doker et al., 2014; Hogan et al., 2015; Jilani et al., 2016; Limmathurotsakul et al., 2016). The current consensus is that the number of melioidosis cases globally is likely grossly under reported, due in part to non-specific signs and symptoms resulting in difficulty with accurate diagnoses (Cheng and Currie, 2005; Peacock, 2006; Wiersinga et al., 2006, 2012). In addition, this bacterium is known to be intrinsically resistant to several commonly used antibiotics, making effective treatment, which results in complete eradication of the bacteria, challenging (Moore et al., 1999; Wuthiekanun et al., 2005; Sarovich et al., 2012). *B. pseudomallei* can cause infections through cutaneous abrasions and lacerations, consumption of contaminated food or drinking water, and is known to be transmitted through an aerosolization process attributable to weather patterns, typically during monsoon season (Inglis et al., 2000; Currie and Jacups, 2003; Currie et al., 2010; Hassan et al., 2010; Limmathurotsakul et al., 2014b; Liu et al., 2015; Kaestli et al., 2016; Sanchez-Villamil et al., 2020).

*Burkholderia pseudomallei* has been a bacterium of concern to the United States Department of Defense for several reasons. It has been demonstrated that healthy individuals can be infected with *B. pseudomallei* and the bacteria can remain undetected for years or decades (Mays and Ricketts, 1975; Koponen et al., 1991; Ngauy et al., 2005). During the Vietnam conflict it was hypothesized that many U.S. military personnel would potentially be exposed to this bacterium while deployed to endemic areas which could result in disease as exposed individuals aged or developed co-morbidities (Patterson et al., 1967; Gilbert et al., 1968; Whelan et al., 1968; Greenberg, 1969; Koponen et al., 1991). Fortunately, the concerns regarding large numbers of latent or unidentified infections in personnel were never realized, however, this remains a concern for deployed individuals (Schully et al., 2019; Larson et al., 2020). Until recently, melioidosis in the U.S. has only been associated with foreign travel or exposure to imported exotic pets (Currie, 2003; Zehnder et al., 2014). However, in 2021 several fatal cases of melioidosis were associated with contaminated aromatherapy liquid manufactured in India and sold in the U.S. at a large national retailer (Centers for Disease Control and Prevention [CDC], 2021; Gee et al., 2022). Thus, this bacterium warrants further attention by both the public health and biodefense research communities.

There has been considerable progress in *B. pseudomallei* vaccine research within the last decade (Limmathurotsakul et al., 2015). Several laboratories have demonstrated successful immunization approaches in both mouse and non-human primate models of disease. These experimental vaccine strategies have included outer membrane vesicles (OMV) (Nieves et al., 2011, 2014; Petersen et al., 2014), live attenuated vaccine strains (Silva et al., 2013; Amemiya et al., 2019; Khakhum et al., 2019a,b), and subunit vaccines consisting of recombinant protein and/or polysaccharide conjugates (Hara et al., 2009; Burtnick et al., 2012, 2018). The vaccines used in this current study include (1) the recombinant proteins alkyl hydroperoxide reductase (AhpC) (Loprasert et al., 2003; Zhang et al., 2019; Schmidt et al., 2022) and/or hemolysin coregulated protein 1 (Hcp1) (Chieng et al., 2015; Lim et al., 2015; Sengyee et al., 2021) combined with the capsular polysaccharide (CPS) conjugated to Cross-Reactive-Material-197 (CRM197) and formulated with Alhydrogel and CpG as adjuvants (Scott et al., 2014) and (2) a live attenuated vaccine strain constructed in the *B. pseudomallei* strain MSHR668 with a deleted *ilvI* gene resulting in a strain that is a branched chain amino acid auxotroph (Atkins et al., 2002; Amemiya et al., 2019). These vaccine strategies have been extensively characterized in mouse models of disease including immune response generated and the protection afforded to the mice after exposure to aerosolized *B. pseudomallei*. Importantly, these vaccines were examined for their ability to induce sterile immunity. Here we report the improvement of disease outcome observed when suboptimal antibiotic regimens were used in combination with current experimental vaccines. A detailed description of the vaccine candidates used in this report and their resulting immune responses is described by Biryukov et al. (2022) within this special issue of Frontiers in Microbiology.

The current antibiotic regimen recommended by the U.S. Centers for Disease Control and Prevention based upon the Darwin melioidosis treatment guidelines consists of at least 2 weeks of intravenous antibiotics (ceftazidime administered every 6–8 h or meropenem administered every 8 h) followed by 3–6 months of oral antimicrobial therapy (co-trimoxazole taken every 12 h or amoxicillin/clavulanic acid taken every 8 h) (Pitman et al., 2015; Sullivan et al., 2020). These guidelines also recommend intravenous antibiotic administration for up to 8 weeks depending upon the extent of infection. For example, Sullivan et al. (2020) recommend 2 weeks of intravenous treatment if a patient presents with unilobar pneumonia but not bacteremia. However, 4 weeks of intravenous antibiotic treatment is recommended for patients exhibiting both bacteremia and pneumonia. Six weeks and 8 weeks of intravenous antibiotic treatment is recommended for cases involving osteomyelitis or central nervous system involvement, respectively. It is important to note that at times abiding by these guidelines may be difficult or impossible due to different medical standards and economic considerations in many areas of the world where *B. pseudomallei* is endemic. The stringency of the melioidosis treatment guidelines underscores the complexity of the bacterial pathogenesis, the non-uniformity of disease progression, and the difficulty associated with disease eradication.

In this report we detail several studies that demonstrate the utility of a combination of medical countermeasures in the C57BL/6 mouse model of inhalational *B. pseudomallei* infection. Antibiotic therapy can significantly augment the protection afforded by vaccines currently in development for the prevention of melioidosis.

## Materials and methods

### Mouse challenge models

C57BL/6 female mice (approximately 7–9 weeks at time of vaccination) were purchased from Charles River (Frederick, MD, United States). In this study we used only female mice. We have previously shown that female and male mice are similar regarding disease course but females are preferred for long course studies due to aggression patterns and self-injury in male mice that often require early euthanasia intervention (Klimko et al., 2020). For challenge studies mice were exposed to aerosolized *B. pseudomallei* K96243 on day 32 (±3) post-last vaccination, as described previously (Bearss et al., 2017; Trevino et al., 2018). Except as identified in footnotes included in tables, all groups of mice had *N* = 10. Briefly, *B. pseudomallei* K96243 was grown overnight in 4% glycerol (Sigma Aldrich, St. Louis, MO, United States), 1% tryptone (Difco, Becton Dickinson, Sparks, MD, United States), and 5% NaCl (Sigma Aldrich) broth (GTB) at 37°C and shaking at 200 rpm. Cultures were harvested by centrifugation and resuspended in fresh GTB medium prior to aerosolization. During the aerosolization procedures an all-glass impinger (AGI) was used to sample aerosolized material so that approximations of inhaled doses could be determined. The challenge doses for each cohort are described in the results. Early endpoint euthanasia was employed in accordance with previously approved intervention criteria. Research was conducted under an IACUC approved protocol in compliance with the Animal Welfare Act, PHS Policy, and other Federal statutes and regulations relating to animals and experiments involving animals. The facility where this research was conducted is accredited by the Association for Assessment and Accreditation of Laboratory Animal Care International and adheres to principles stated in the Guide for the Care and Use of Laboratory Animals, National Research Council, 2011.

At the end of study, surviving mice were euthanized and examined for evidence of detectable pyogranulomas during gross necropsy. After euthanasia, the lungs and spleens were removed, weighed, and homogenized for colony forming unit (CFU) determination to approximate bacterial load present in each organ. Homogenates were plated on sheep blood agar plates (Remel ThermoFisher, Rockville, MD, United States) and incubated at 37°C for 48 h. The limit of detection for each organ was determined to be approximately 5 CFU per organ based upon the homogenization and plating protocol used.

### Vaccination strategies

Vaccinations were carried out as previously described (Burtnick et al., 2012, 2018; Amemiya et al., 2019; Biryukov et al., 2022). Briefly, mice receiving the live attenuated vaccine 668 Δ*ilvI* were vaccinated on day 0 and day 21 or 24 with a target dose of approximately 1.0 × 10^7^ CFU delivered in 200 μl injections subcutaneously. Mice described in Table 1 received a prime vaccine dose of approximately 8.6 × 10^6^ CFU on day 0 and then a booster dose of approximately 9.8 × 10^6^ CFU on day 21. Mice described in Tables 2, 3 received a prime vaccine dose of 8.0 × 10^6^ CFU on day 0 and then a booster dose of approximately 1.4 × 10^6^ CFU on day 24. Mice receiving subunit vaccines were vaccinated three times (day 0, day 21, and day 35 for the experiment listed in Table 1 and day 0, day 21, and day 38 for the experiments listed in Tables 2, 3). Each dose of the subunit vaccine included 0.5 μg of the recombinant proteins listed: enzymatically inactive AhpC; (Schmidt et al., 2022) and/or tagless Hcp1; (Burtnick MN, unpublished), 0.25 μg of the CPS conjugated to CRM197 (conjugate) (Fina Biosolutions, Rockville, MD, United States); 250 μg of Alhydrogel (Invivogen, San Diego, CA, United States), and 10 μg of CpG ODN 2006 (Invivogen). The subunit vaccines were also delivered in 200 μl doses, but with 100 μl being delivered to each hind flank. In general, the mice responded well to all vaccines administered; however, in some cases significant reactogenicity (most often associated with the administration of the vaccine formulation containing AhpC) resulted in several mice being removed from the study before exposure to aerosolized *B. pseudomallei*. The experimental groups that had mice removed from study are noted as footnotes in the data tables. Submandibular blood collections were taken approximately 1 week prior to challenge to assess the immune response in the mice prior to exposure to aerosolized *B. pseudomallei*. ELISA-derived antibody titers against select antigens are provided in the Supplementary Table 1.

**TABLE 1:** Vaccinated or naïve C57BL/6 mice are similarly protected after receiving 21 days of co-trimoxazole initiated within 45 h after exposure to aerosolized *B. pseudomallei*. Vaccinated mice were more likely to survive than naive mice when co-trimoxazole was initiated 69 h post-exposure.

Vaccination status and treatment groups^a^	% Survival through day 30 post challenge	Day 30 survival *P*-value^b^	% Survival through day 82 post challenge	Day 82 Survival *P*-value^b^	Ratio of Sterile survivors/Total Survivors^c^	TTM *P*-value^b^
PBS	0%	n/a	0%	n/a	n/a	n/a
PBS + co-trimoxazole 21 h	100%	<0.0001	90%	<0.0001	9/9	<0.0001
PBS + co-trimoxazole 45 h	100%	<0.0001	100%	<0.0001	8/10^e^	<0.0001
PBS + co-trimoxazole 69 h	40%	NS^d^	30%	NS	2/3^e^	NS
668 Δ*ilvI*	20%	NS	20%	NS	0/2^e^	0.0002
668 Δ*ilvI* + co-trimoxazole 21 h	100%	0.0007	100%	0.0007	10/10	0.0003
668 Δ*ilvI* + co-trimoxazole 45 h	100%	0.0007	90%	0.0007	9/9	0.0008
668 Δ*ilvI* + co-trimoxazole 69 h	90%	0.0055	90%	0.0055	9/9	0.0024
AhpC + Hcp1 + conjugate	60%	0.0108	60%	0.0108	5/6^e^	<0.0001
AhpC + Hcp1 + conjugate + co-trimoxazole 21 h	100%	NS	100%	NS	10/10	0.0291
AhpC + Hcp1 + conjugate + co-trimoxazole 45 h	100%	NS	100%	NS	10/10	0.0291
AhpC + Hcp1 + conjugate + co-trimoxazole 69 h	100%	NS	90%	NS	9/9	NS

^a^Mice were vaccinated as described in Section “Materials and methods.” If co-trimoxazole was provided it was initiated at the time-point after exposure to approximately 2.86 × 10^3^ CFU (+/− 3.95 × 10^2^ CFU) of aerosolized *B. pseudomallei* K96243 on day 29 post-last vaccination.

^b^Statistical comparison to respective control group without co-trimoxazole or in the case of vaccines only to the PBS alone group. *P*-values indicate the result of a Log-rank or Fisher exact test for the survival and TTM, respectively.

^c^Survivors were determined to be free of *B. pseudomallei* CFU in lungs and spleens and there was no visible pyogranuloma formation noted at gross necropsy.

^d^NS, not significant.

^e^See Supplementary Table 2 for bacterial burden data for each mouse shown to retain B. pseudomallei. Limit of detection for tissue homogenates is 5 CFU/organ.

**TABLE 2:** Vaccinated or naïve C57BL/6 mice are similarly protected after receiving 7 days of co-trimoxazole initiated within 45 h after exposure to aerosolized *B. pseudomallei*. Only vaccinated mice survived when co-trimoxazole was initiated 69 h post-exposure.

Vaccination status and treatment groups^a^	% Survival through day 30 post challenge	Day 30 survival *P*-value^b^	% Survival through day 70 post challenge	Day 70 survival *P*-value^b^	Ratio of Sterile survivors/Total Survivors^c^	TTM *P*-value^b^
PBS	0%	n/a	0%	n/a	n/a	n/a
PBS + co-trimoxazole 9 h	100%	<0.0001	100%	<0.0001	10/10	<0.0001
PBS + co-trimoxazole 21 h	100%	<0.0001	90%	0.0001	6/9^e^	0.0001
PBS + co-trimoxazole 45 h	70%	0.0031	70%	0.0031	6/7^e^	0.0002
PBS + co-trimoxazole 69 h	0%	NS^d^	0%	NS	n/a	NS
668 Δ*ilvI*	20%	NS	20%	NS	1/2^e^	<0.0001
668 Δ*ilvI* + co-trimoxazole 9 h	100%	0.0007	90%	0.0055	10/10	0.0007
668 Δ*ilvI* + co-trimoxazole 21 h	100%	0.0007	100%	0.0007	10/10	0.0003
668 Δ*ilvI* + co-trimoxazole 45 h	100%	0.0007	100%	0.0007	7/10^e^	0.0003
668 Δ*ilvI* + co-trimoxazole 69 h	90%	0.0055	80%	0.0230	6/8^e^	0.0017
AhpC + Hcp1 + conjugate^f^	56%	0.0325	22%	NS	2/2	<0.0001
AhpC + Hcp1 + conjugate + co-trimoxazole 9 h^f^	100%	0.0294	100%	0.0023	9/9	0.0008
AhpC + Hcp1 + conjugate + co-trimoxazole 21 h^g^	100%	0.0294	100%	0.0023	8/8	0.0015
AhpC + Hcp1 + conjugate + co-trimoxazole 45 h^f^	100%	0.0294	100%	0.0023	8/9^e^	0.0008
AhpC + Hcp1 + conjugate + co-trimoxazole 69 h^f^	100%	0.0294	89%	0.0152	8/9^e^	0.0020
Hcp1 + conjugate	60%	0.0108	20%	NS	2/2	<0.0001
Hcp1 + conjugate + co-trimoxazole 9 h^f^	100%	NS	100%	0.0007	9/9	0.0005
Hcp1 + conjugate + co-trimoxazole 21 h	90%	NS	90%	0.0055	9/9	0.0031
Hcp1 + conjugate + co-trimoxazole 45 h	100%	NS	100%	0.0007	9/10^e^	0.0003
Hcp1 + conjugate + co-trimoxazole 69 h^f^	100%	NS	100%	0.0007	8/9^e^	0.0005

Only vaccinated mice survived the infection when co-trimoxazole was initiated at 69 h post exposure.

^a^Mice were vaccinated as described in Section “Materials and methods.” If co-trimoxazole was provided it was initiated at the time-point after exposure to approximately 2.45 × 10^3^ CFU (+/− 4.33 × 10^2^ CFU) of aerosolized *B. pseudomallei* K96243 on day 35 post-last vaccination.

^b^Statistical comparison to respective control group without co-trimoxazole or in the case of vaccines only to the PBS alone group. *P*-values indicate the result of a Log-rank or Fisher exact test for the survival and TTM, respectively.

^c^Survivors were determined to be free of *B. pseudomallei* CFU in lungs and spleens and there was no visible pyogranuloma formation noted at gross necropsy.

^d^NS, not significant.

^e^See Supplementary Table 2 for bacterial burden data for each mouse shown to retain *B. pseudomallei*. Limit of detection for tissue homogenates is 5 CFU/organ.

^f^*N* = 9.

^g^*N* = 8.

**TABLE 3:** Only vaccinated mice survived the infection if 21 days of co-trimoxazole was initiated 93 h or 117 h post exposure to aerosolized *B. pseudomallei*.

Vaccination status and treatment groups^a^	% Survival through day 30 post challenge	Day 30 survival *P*-value^b^	% Survival through day 86 post challenge	Day 86 survival *P*-value^b^	Ratio of Sterile survivors/Total Survivors^c^	TTM *P*-value^b^
PBS	0%	n/a	0%	n/a	n/a	n/a
PBS + co-trimoxazole 93 h	0%^f^	NS^d^	0%	NS	n/a	NS
PBS + co-trimoxazole 117 h	0%^g^	NS	0%	NS	n/a	0.0018
668 Δ*ilvI*	30%	NS	30%	NS	3/3	<0.0001
668 Δ*ilvI* + co-trimoxazole 93 h	100%	0.0031	100%	0.0031	9/10^e^	0.0012
668 Δ*ilvI* + co-trimoxazole 117 h	100%	0.0031	100%	0.0031	9/10^e^	0.0012
AhpC + Hcp1 + conjugate	80%	0.0007	40%	NS	4/4	<0.0001
AhpC + Hcp1 + conjugate + co-trimoxazole 93 h	100%	NS	100%	0.0108	9/10^e^	0.0039
AhpC + Hcp1+ conjugate + co-trimoxazole 117 h	100%	NS	100%	0.0108	9/10^e^	0.0039
Hcp1 + conjugate	80%	0.0007	70%	0.0031	5/7^e^	<0.0001
Hcp1 + conjugate + co-trimoxazole 93 h	100%	NS	100%	NS	7/10^e^	NS
Hcp1 + conjugate + co-trimoxazole 117 h	90%	NS	90%	NS	9/9	NS

^a^Mice were vaccinated as described in Section “Materials and methods.” If co-trimoxazole was provided it was initiated at the time-point after exposure to approximately 1.57 × 10^3^ CFU (+/− 2.34 × 10^2^ CFU) of aerosolized *B. pseudomallei* K96243 on day 35 post-last vaccination. Sham vaccinated mice were not rescued from lethal infection with the delayed initiation of co-trimoxazole therapy.

^b^Statistical comparison to respective control group without co-trimoxazole or in the case of vaccines only to the PBS alone group. *P*-values indicate the result of a Log-rank or Fisher exact test for the survival and TTM, respectively.

^c^Survivors were determined to be free of *B. pseudomallei* CFU in lungs and spleens and there was no visible pyogranuloma formation noted at gross necropsy.

^d^NS, not significant.

^e^See Supplementary Table 2 for bacterial burden data for each mouse shown to retain *B. pseudomallei*. Limit of detection for tissue homogenates is 5 CFU/organ.

^f^50% of mice succumbed or were euthanized prior to co-trimoxazole initiation.

^g^100% of mice succumbed or were euthanized prior to co-trimoxazole initiation.

### Antibiotic regimens

The antibiotic chosen for this study was co-trimoxazole (Teva Pharmaceuticals USA Inc., North Wales, PA, United States) diluted to achieve a dose of approximately 100 mg/kg of sulfamethoxazole USP and 20 mg/kg of trimethoprim USP. The co-trimoxazole was diluted in 5% dextrose in water. Control animals (no antibiotics) received 5% dextrose in water alone. Antibiotics were delivered *via* intraperitoneal injections every 12 h for either 7 or 21 days as described in the data tables.

### Enzyme-linked immunosorbent assay analyses

Immunoglobulin G (IgG) titers in vaccinated mice were determined by ELISA as described by Trevino et al. (2018). The capture reagents included capsule CPS and whole-cell radiation-inactivated *B*. *pseudomallei* K96243 cells (BpK). The antibody titer results obtained from the pooled sera samples are reported as the geometric mean (GM) and geometric standard error (GSE) of the reciprocal of the highest dilution giving a mean OD of at least 0.100 ± 1 SD at 450 nm with a reference filter (570 nm). The limit of detection was a reciprocal titer of 50 and samples with an antibody titer of <50 were considered negative. The labeled secondary antibody used in the ELISAs was goat anti-mouse IgG obtained from Southern Biotechnology Associates, Inc. (Birmingham, AL, United States).

### Statistics

The survival rates at selected time points were compared by Fisher exact test and the times to morbidity (TTM) were analyzed by Log-rank test. Where feasible, the potential synergy between antibiotic and vaccine was analyzed by forming a test of interaction in a log-logistic accelerated failure time model. The synergy score is the fold increase in survival time associated with vaccination in the antibiotic treated animals, divided by the fold increase in survival time associated with vaccination in the absence of antibiotic treatment. A Wald test was used to compare the synergy score to 1. Analysis was implemented in SAS version 9.4 (SAS Institute Inc., Carry, NC, United States).

## Results

### Extended co-trimoxazole regimen initiated early after exposure to aerosolized *Burkholderia pseudomallei* results in significant protection of C57BL/6 mice

As shown in Table 1, when 21 days of co-trimoxazole is initiated within 45 h after naïve mice are exposed to aerosolized *B. pseudomallei* (approximately 7 LD_50_ equivalents) the survival rates 82 days post-exposure to *B. pseudomallei* are 90% or greater. Mice that received the co-trimoxazole starting within 21 h demonstrated 100% sterile immunity and if the co-trimoxazole was initiated at 45 h post-exposure, 80% of the mice were determined to be free of *B. pseudomallei* (as determined by culturing of lung, spleen, and blood). If treatment was delayed to approximately 69 h post-exposure to *B. pseudomallei*, the survival rate was reduced to 30% and sterility was observed in two out of three surviving mice.

As previously discussed, several current promising *B. pseudomallei* vaccine candidates have been characterized. These vaccines include protein subunit conjugate vaccines and live attenuated vaccines, and they result in various antibody titers (Supplementary Table 1) and levels of protection after exposure to aerosolized *B. pseudomallei*. We first examined the protein subunit vaccine consisting of AhpC, Hcp1, CPS conjugated to CRM197, Alhydrogel, and CpG as well as the live attenuated vaccine strain 668 Δ*ilvI.* In this experiment, the protein subunit vaccine, and the vaccine strain 668 Δ*ilvI* resulted in 60% and 20% protection at the end of study, respectively (Table 1). However, when the vaccinated mice were also treated with co-trimoxazole after exposure to aerosolized *B. pseudomallei* the survival rates were 90% or greater and all surviving mice were determined to be free from infection with *B. pseudomallei*. The layering of the medical countermeasures resulted in statistically significant improvement in the survival rates and time to morbidity (TTM) compared to vaccinated mice with no post-exposure antibiotic treatment (Table 1). While promising, this early initiation of treatment coupled with an extended antibiotic dosing schedule resulted in data that did not demonstrate significant synergy between the vaccines and antibiotic regimen because co-trimoxazole alone rescued most of the non-vaccinated mice and only three survivors had detectable bacteria in the lungs (Supplementary Table 2).

### Vaccinated mice are protected with shorter duration or delayed courses of co-trimoxazole

**FIGURE 1:**
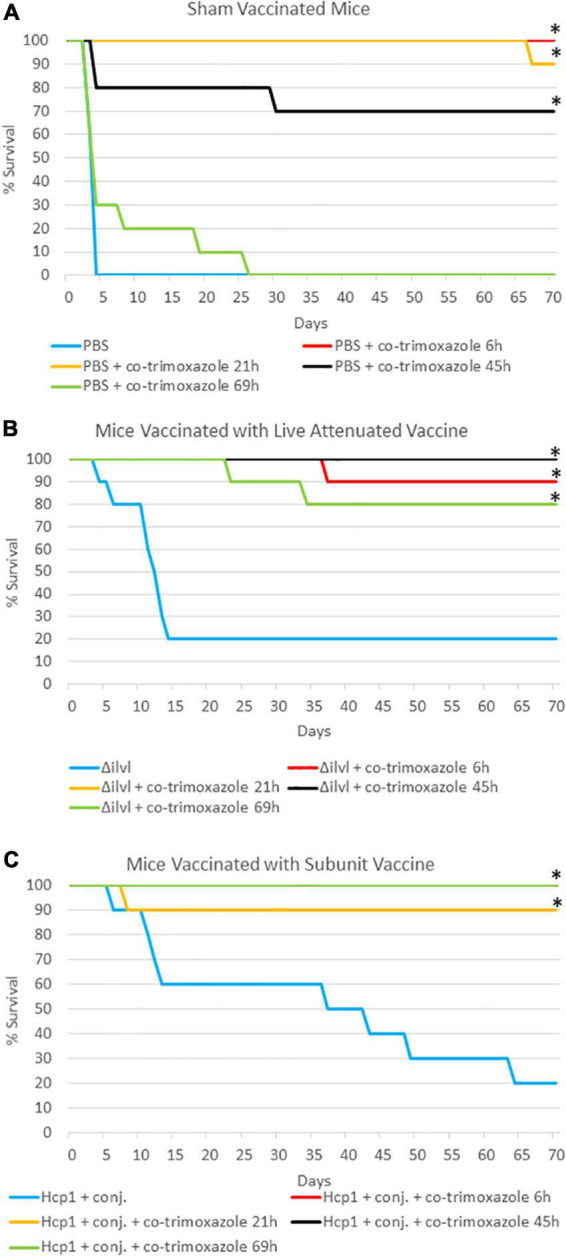
The impact of combining prophylactic vaccine with a short course (7 days) of post-exposure co-trimoxazole therapy initiated early after exposure to aerosolized *B. pseudomallei*. **(A)** Mice were vaccinated with sham vaccine (PBS) before exposure to *B. pseudomallei* K96243. **(B)** Mice were vaccinated with the live attenuated vaccine 668 Δ*ilvI* before exposure to *B. pseudomallei* K96243 (21 h data are not visible; all mice survived through the end of study). **(C)** Mice were vaccinated with the subunit Hcp1 + conjugate vaccine before exposure to *B. pseudomallei* K96243 (6 h and 45 h data are not visible; all mice survived through the end of study). Antibiotics were initiated at the time indicated and were given every 12 h for 7 days. These data are representative graphic depiction of data included in Table 2; *P < 0.05 for survival compared to vaccine alone, actual *P*-values are indicated in Table 2.

To more stringently evaluate the extent of the protection co-trimoxazole provided vaccinated mice, we performed two additional experiments. As described in Table 2 and Figure 1, naïve or vaccinated mice were exposed to approximately 6 LD_50_ equivalents of aerosolized *B. pseudomallei* K96243 followed by a shortened 7 day, rather than a 21-day, time course of antibiotics post-challenge. In this experiment, results were similar to those obtained from the cohort that received co-trimoxazole for 21 days (Table 1), with the exception being that the shortened time course offered an opportunity to demonstrate statistically significant synergy between the vaccines and antibiotic therapy. Among animals not treated by co-trimoxazole, modeling of survival times found an estimated 5.7-fold and 3.7-fold increase in survival times in mice immunized with AhpC + Hcp1 + conjugate or 668 Δ*ilvI*, respectively. By comparison, treatment with co-trimoxazole at 69 h gave improvements in survival times of 40.4- and 26.8-fold in these two immunized groups. The increase in the relative effect of these vaccines was statistically significant (*P* < 0.01 by Wald test) in each case and gives evidence of a synergistic effect. The high levels of survival did not permit similar quantification of the synergy in each immunization group, respectively. However, vaccinated mice that received the antibiotics in this delayed regimen demonstrated 80% or greater survival rates and only a few mice retained low levels of *B. pseudomallei* in the lungs and spleens (Supplementary Table 2).

**FIGURE 2:**
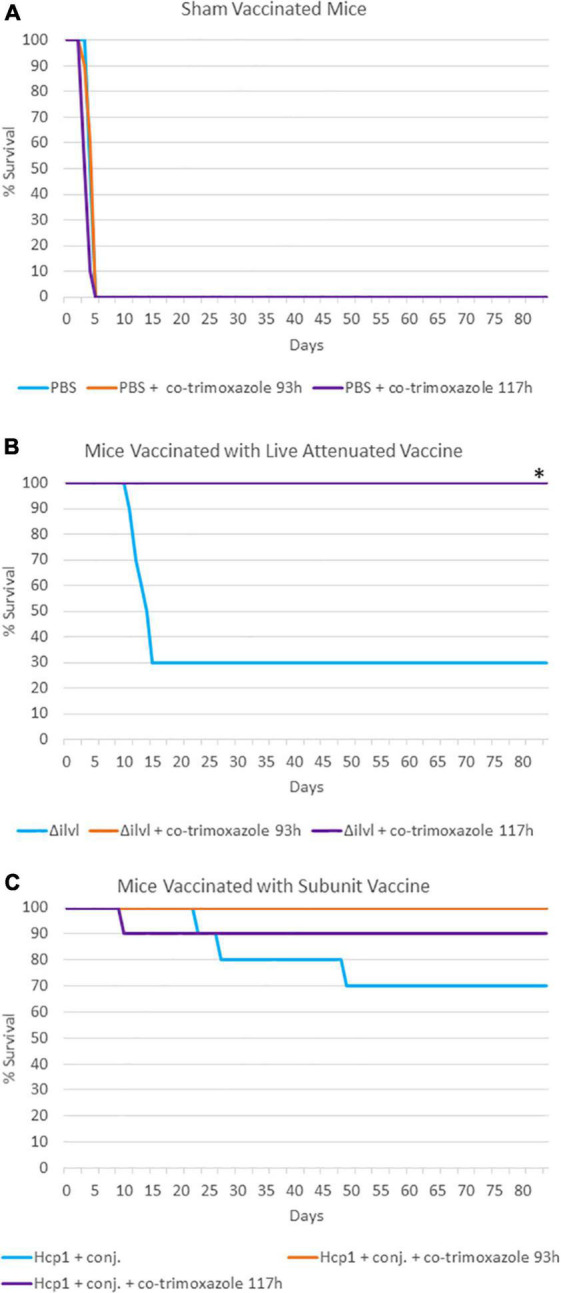
The impact of combining prophylactic vaccine with a long course (21 days) of post-exposure co-trimoxazole therapy with a delayed initiation after exposure to aerosolized *B. pseudomallei*. **(A)** Mice were vaccinated with sham vaccine (PBS) before exposure to *B. pseudomallei* K96243. **(B)** Mice were vaccinated with the live attenuated vaccine 668 Δ*ilvI* before exposure to *B. pseudomallei* K96243 (93 h data are not visible; all mice survived through the end of study). **(C)** Mice were vaccinated with the subunit Hcp1 + conjugate vaccine before exposure to *B. pseudomallei* K96243. Antibiotics were initiated at the time indicated and were given every 12 h for 21 days. These data are representative graphic depiction of data included in Table 3; *P < 0.05 for survival compared to vaccine alone, actual *P*-values are indicated in Table 3.

A third experiment, summarized in Table 3 and Figure 2, evaluated naïve or vaccinated mice that were exposed to approximately 4 LD_50_ equivalents of aerosolized *B. pseudomallei* K96243 and were then treated with a 21-day antibiotic regimen with a delayed initiation time (approximately 93 or 117 h post-challenge). With these infection and treatment parameters, none of the naïve mice survived the challenge (all succumbed or were euthanized within 21 days). Importantly, in this experiment, 50% of the non-vaccinated mice succumbed or were euthanized prior to the initiation of co-trimoxazole at 93 h post-exposure to *B. pseudomallei* and 100% of the non-vaccinated mice succumbed or were euthanized prior to the initiation of co-trimoxazole at 117 h post-exposure to *B. pseudomallei*. Vaccinated mice that received the antibiotics in this delayed regimen demonstrated 90% or greater survival rates and only a few mice retained low levels of *B. pseudomallei* in the lungs and spleens (Supplementary Table 2).

## Discussion

The data described here provide proof-of-concept that partially protective *B. pseudomallei* vaccination strategies can synergize with suboptimal antibiotic regimens resulting in nearly 100% survival in a C57BL/6 mouse model of melioidosis. The concept of combining medical countermeasures in hopes of achieving synergism is not new. Benefits of combining different medical countermeasures have been documented or hypothesized, including (but not limited to) combinations of subunit vaccines and antibiotics (Vietri et al., 2006; Klinman and Tross, 2009; Klinman et al., 2009; Brady et al., 2011; Leffel et al., 2012), combinations of chemotherapy with immunotherapy (Stevens, 1998; Rodrigues et al., 2015; Jones et al., 2018; Ramamurthy et al., 2021), combinations of antibiotic and antimicrobial peptides (Almaaytah et al., 2018; Zharkova et al., 2019; Cote et al., 2020), combinations of monoclonal antibodies delivered as a cocktail or in combination with immunization (Hill et al., 2003; Casadevall et al., 2004; Grabenstein, 2008; Diamant et al., 2015; Gilchuk et al., 2020; Liang et al., 2021), combinations of monoclonal antibodies and antibiotics (Buchwald and Pirofski, 2003; Biron et al., 2015; Migone et al., 2015; Domenech et al., 2018), and combinations of antibiotics and phage therapies (Abedon, 2019; Li et al., 2021; Wang et al., 2021).

In the case of *Bacillus anthracis* infection, improvement has been observed when combining medical countermeasures. In a post-exposure scenario, Vietri et al. (2006) demonstrated significant improvement if non-human primates that were exposed to aerosolized *B. anthracis* spores were treated with ciprofloxacin in combination with three doses of the AVA anthrax vaccine as compared to animals only receiving ciprofloxacin. A similar impact of combining a single dose of dalbavancin and the AVA vaccine adjuvanted with CpG was demonstrated using a mouse model of anthrax (Klinman and Tross, 2009). More recently, an appreciable (but not statistically significant) benefit was reported using a rabbit model of anthrax that received levofloxacin with polyclonal immunoglobulin therapy (Kammanadiminti et al., 2014) or in rabbits treated with monoclonal antibody therapy in combination with levofloxacin (Migone et al., 2015). However, statistical significance was achieved when monoclonal antibody therapy was combined with doxycycline treatment in rabbits exposed to aerosolized *B. anthracis* spores (Biron et al., 2015). These are representative layered approaches for the treatment of anthrax; however, the rather complex pathogenesis of *B. anthracis* (e.g., bacterial spore infectivity, spore germination, intoxication of host, and severely acute disease with near uniform lethality) may not be reflective of other bacterial infections.

Perhaps a more representative example of potential synergy observed when layering medical countermeasures is *Staphylococcus aureus* infection. Using a rabbit model of biofilm formation associated with osteomyelitis, immunizations alone resulted in a reduction in clinical signs associate with *S. aureus*-induced osteomyelitis; however, the animals retained significant bacterial burden in the bone tissue. When vaccinated animals received vancomycin post-challenge, there was a significant reduction in bacteria and an enhanced clearance rate that was only observed in the cohort of animals receiving prophylactic vaccination in combination with post-exposure antibiotics (Brady et al., 2011).

There are several factors that contribute to the difficulty of treating patients with melioidosis with a single medical countermeasure; for example, this bacterium is difficult to treat because of intrinsic antibiotic resistance. Additionally, the clinical presentation of *B. pseudomallei* infection is extremely diverse and can include abscess/pyogranulomas in multiple organs, osteomyelitis, and primary or secondary pneumonia (Currie, 2003; Teparrakkul et al., 2008; Morse et al., 2009; Currie et al., 2010, 2021; Meumann et al., 2012; Raja and Scarsbrook, 2016; Kozlowska et al., 2018; Soo et al., 2021; Wu et al., 2021). Mouse models have partially recapitulated some of the diverse clinical manifestations of human melioidosis. BALB/c mice are considered a model for acute melioidosis, while C57BL/6 mice are less susceptible to *B. pseudomallei* and tend to develop characteristics that are like chronic forms of melioidosis (Leakey et al., 1998; Liu et al., 2002; Titball et al., 2008; Conejero et al., 2011; Welkos et al., 2015).

In the absence of distinct clinical manifestations, many cases of infection with *B. pseudomallei* are difficult to diagnose and the disease is often referred to as the “great mimicker.” Patients in endemic areas can be infected multiple times (or by multiple strains in rare instances) and individuals traveling to endemic areas have been documented to have no disease presentation until years or decades after the primary encounter with the bacterium (Mays and Ricketts, 1975; Koponen et al., 1991; Ngauy et al., 2005; Limmathurotsakul et al., 2007; Pitt et al., 2007). Further complicating treatment plans are the known risk factors for susceptibility to *B. pseudomallei* infection (e.g., diabetes and heavy alcohol consumption) and the disparate quality of medical care available in some geographic locations where *B. pseudomallei* is known to be or suspected to be endemic (Hassan et al., 2010). Consequently, we believe *B. pseudomallei* infections will be optimally treated with layered and integrated medical countermeasures.

Significant effort has been spent on the identification, characterization, and optimization of protective vaccine antigens that will prevent or ameliorate *B. pseudomallei* infections and melioidosis disease. Various vaccine strategies including live attenuated vaccine strains, OMVs, and protein subunit/polysaccharide conjugate combinations have produced robust immune responses that have been protective in both mouse and non-human primate models of melioidosis (Atkins et al., 2002; Nieves et al., 2011, 2014; Burtnick et al., 2012, 2018; Petersen et al., 2014; Scott et al., 2014; Titball et al., 2017; Amemiya et al., 2019; Khakhum et al., 2019a; Biryukov et al., 2022). Even with these successes in vaccine strategies, there remains the possibility that survivors of the acute phase of the infection could continue to harbor the bacteria that could reemerge later. There has been equally impressive progress toward the understanding and evaluation of antibiotic regimens that are protective (Sullivan et al., 2019, 2020). However, diagnosed *B. pseudomallei* infections continue to warrant extended intravenous antibiotic regimen followed by oral antibiotics for months (Pitman et al., 2015; Sullivan et al., 2020). In our studies, we chose to use co-trimoxazole as the antibiotic paired with our vaccines in C57BL/6 mice. This antibiotic has been previously shown to be successful in the more susceptible BALB/c mouse models of melioidosis (Ulett et al., 2003; Sivalingam et al., 2008; Barnes et al., 2013, 2017) and continues to be an important component for patient treatment plans (Sullivan et al., 2019, 2020).

In the C57BL/6 mouse model of *B. pseudomallei* infection, combining vaccination strategies with antibiotic regimens demonstrated a clear advantage over mice that were only vaccinated or mice that only received co-trimoxazole. In our first iteration, we initiated co-trimoxazole, given every 12 h, starting at approximately 21, 45, or 69 h post exposure to aerosolized *B. pseudomallei* for 21 days (Table 1). Treatment with co-trimoxazole alone at 69 h post-infection only protected 30% of the animals. However, if vaccinated mice were treated with co-trimoxazole starting at 69 h post-infection, 90% of the mice were protected through day 82.

To test the limits of this combination therapy and to examine if the protection afforded by vaccines could allow for a shortened antibiotic treatment course, the co-trimoxazole treatment of 21 days was reduced to 7 days and the treatment was initiated at time points ranging from approximately 9–69 h post-infection. In the absence of vaccination, no mice survived the infection after the shortened course of co-trimoxazole when the treatment was initiated 69 h post-exposure (Table 2), however, vaccinated mice receiving co-trimoxazole treatment starting at 69 h post-exposure led to 80% or greater survival through day 70. Finally, we tested a 21-day co-trimoxazole regimen but with a delayed initiation at either approximately 93 h or 117 h post-infection. As described in Figure 2 and Table 3, using this antibiotic regimen and under these experimental conditions, uniform lethality occurred by day 5 post-exposure. However, vaccinated mice receiving these delayed antibiotic treatments exhibited 90% or greater survival rates and most of the mice surviving through day 85 had no evidence of residual bacteria in lungs or spleens. It is important to note that we only sampled the animals that survived through the end of study for bacterial burden and did not perform a serial sampling experiment. Additionally, it is imperative to reiterate that we only sampled spleens and lungs and examined the animals for gross signs of pyogranuloma formation in other tissues. Due to the significant heterogeneity of clinical presentation of melioidosis and the propensity of *B. pseudomallei* to enter a persister or non-culturable state (Pumpuang et al., 2011; Li et al., 2014; Lewis and Torres, 2016), we cannot verify complete sterility in the surviving animals.

Once proven safe and immunogenic, licensed *B. pseudomallei* vaccines should be encouraged in endemic areas with high clinical incidence. These vaccination strategies will, at the very least, ameliorate the acute form of the disease, but possibly and perhaps more importantly allow for shorter courses of effective antibiotics or provide larger windows of opportunity to initiate effective antibiotic therapy. In addition to improved patient outcomes, the combinatorial strategies of vaccines and antibiotics offer the secondary advantage of potentially reducing the emergence of antimicrobial resistant isolates. There is significant interest and conjecture regarding how vaccines may be used to combat antimicrobial resistance (Lipsitch and Siber, 2016; Klugman and Black, 2018; Antonelli et al., 2021; Micoli et al., 2021; Vekemans et al., 2021). Clinical data have suggested that antibiotic resistance in *B. pseudomallei* could be associated with patients that respond poorly to antibiotic therapy (Fen et al., 2021). The data generated in our mouse models of inhalational melioidosis suggest that vaccinated patients would respond better to antibiotic therapy. Shortened antibiotic durations in vaccinated individuals may be protective and could lead to reduced numbers of antibiotic resistant bacterial isolates (*B. pseudomallei* or normal flora) arising after exposure to prolonged or less-effective antibiotic regimens (Sunde et al., 1998; Vatopoulos et al., 1998; Rice, 2008; Fair and Tor, 2014; Ventola, 2015a,b). Even if a vaccine was partially protective, vaccinated individuals could have lessened clinical symptoms or fewer secondary bacterial infections that would have likely resulted in unnecessary antibiotic regimens (Lewnard et al., 2020; Rodgers et al., 2021). Thus, patient outcomes should be considerably improved if individuals are immunized, and appropriate antibiotic therapy is initiated as soon as possible after possible exposure to *B. pseudomallei.*

Another intriguing concept suggests that vaccines that inhibit biofilm formation could result in a bacterial population more likely to remain in a planktonic state and, accordingly, more susceptible to antibiotic therapy (Sawasdidoln et al., 2010; Brady et al., 2011; Mirzaei et al., 2021a,b). *B. pseudomallei* has been demonstrated to form biofilms in both the laboratory setting and in the lungs of infected animals and humans (Vorachit et al., 1995; Limmathurotsakul et al., 2014a) and is known to generate persister cells that have multiple antibiotic tolerance profiles (Lewis, 2005, 2008; Hamad et al., 2011; Conlon et al., 2015; Nierman et al., 2015; Bernhards et al., 2017).

Ultimately, the most appropriate combination strategies will depend upon the effectiveness of the individual medical countermeasure as well as the severity and time course of disease progression. While combinatorial approaches offer, improved strategies there are reports documenting negative effects, including potential inhibition of vaccine or phage therapy efficacy by the administration of certain antibiotics or monoclonal antibodies, and these possibilities must be considered when designing experiments or putative treatment plans (Woo et al., 1999; Eyles et al., 2007; Abedon, 2019). Lastly, an important advantage of the layered defense strategy is the fact that previously dismissed medical countermeasures may now be able to play an important role in prevention strategies and/or therapeutic approaches. Combining medical countermeasures would provide significantly greater numbers of effective strategies, which are urgently needed when planning for emerging, re-emerging, or potentially engineered bacterial threats in both public health and biodefense arenas.

## Data availability statement

The raw data supporting the conclusions of this article will be made available by the authors, without undue reservation.

## Ethics statement

The animal study was reviewed and approved by United States Army Medical Research Institute of Infectious Diseases (USAMRIID) Institutional Animal Care and Use Committee (IACUC).

## Author contributions

CK, MB, PB, DD, and CC designed and supervised the project. CK, JS, NR, MH, JD, YT, LS, CO, SB, and CC prepared vaccine antigens and performed the experiments. CK, DF, and CC performed data analyses. SB and CC wrote the manuscript. MB, PB, SB, DD, and CC edited the manuscript. All authors contributed to the article and approved the submitted version.
