# Supplementary material for: Layered and integrated medical countermeasures against Burkholderia pseudomallei infections in C57BL/6 mice
Source: Front Microbiol. 2022 Aug 17;13:965572. doi: 10.3389/fmicb.2022.965572 (PMC9432870; doi:10.3389/fmicb.2022.965572)
Supplement: Supplementary file 1 [file Data_Sheet_1.docx]

Table S1. Humoral immune responses generated after vaccination.

Table S2. Bacterial burden in mice that retained *B. pseudomallei* at the end of study.
